# T-Large Granular Lymphocytic Leukemia with Hepatosplenic T-Cell Lymphoma? A Rare Case of Simultaneous Neoplastic T-Cell Clones Highlighted by Flow Cytometry and Review of Literature

**DOI:** 10.3390/biomedicines12050993

**Published:** 2024-04-30

**Authors:** Rossana Libonati, Michela Soda, Teodora Statuto, Luciana Valvano, Fiorella D’Auria, Giovanni D’Arena, Giuseppe Pietrantuono, Oreste Villani, Giovanna Rosaria Mansueto, Simona D’Agostino, Massimo Dante Di Somma, Alessia Telesca, Rocchina Vilella

**Affiliations:** 1 Laboratory of Clinical Research and Advanced Diagnostics, Centro di Riferimento Oncologico della Basilicata (IRCCS-CROB), 85028 Rionero In Vulture, Italy; rossana.libonati@crob.it (R.L.); michela.soda@crob.it (M.S.); alessia.telesca@crob.it (A.T.); rocchina.vilella@crob.it (R.V.); 2 Laboratory of Clinical Pathology, Centro di Riferimento Oncologico della Basilicata (IRCCS-CROB), 85028 Rionero In Vulture, Italy; fiorella.dauria@crob.it; 3 Immunohematology and Transfusional Medicine, “S. Luca” Hospital, ASL Salerno, 84078 Vallo della Lucania, Italy; giovannidarena@libero.it; 4 Hematology and Stem Cell Transplantation Unit, Centro di Riferimento Oncologico della Basilicata (IRCCS-CROB), 85028 Rionero in Vulture, Italy; giuseppe.pietrantuono@crob.it (G.P.); oreste.villani@crob.it (O.V.); giovanna.mansueto@crob.it (G.R.M.); simona.dagostino@crob.it (S.D.); 5 Anatomical Pathology Department, Centro di Riferimento Oncologico della Basilicata (IRCCS-CROB), 85028 Rionero in Vulture, Italy; massimodante.disomma@crob.it

**Keywords:** T-large granular lymphocytic leukemia, hepatosplenic t-cell lymphoma, mixed-phenotype, flow cytometry, clonality

## Abstract

Lymphoproliferative diseases are a heterogeneous set of malignant clonal proliferations of lymphocytes. Despite well-established diagnostic criteria, the diagnosis remains difficult due to their variety in clinical presentation and immunophenotypic profile. Lymphoid T-cell disorders are less common than B-cell entities, and the lack of a clear immunophenotypic characteristic makes their identification hard. Flow cytometry turned out to be a useful tool in diagnosing T-cell disorders and to resolve complicated cases, especially if the number of analyzable neoplastic cells is small. We present a case of a 55-year-old man with simultaneous lymphoproliferative neoplastic T-cell clones, one αβ and the other γδ, identified and characterized by flow cytometry (FC), exploiting the variable expression intensity of specific markers. However, the patient’s rapid decline made it impossible to define a differential diagnosis in order to confirm the identity of the γδ clone, which remains uncertain. This case is added to the few other cases already documented in the literature, characterized by the co-existence of T-large granular lymphocytic leukemia (T-LGLL)-αβ and T-LGLL-γδ/Hepatosplenic T-cell lymphoma (HSTCL). Our case underlines the key role of sensitive diagnostic tools in the assessment of potential relationship between the diagnosis, prognosis, and treatment in the two pathologies.

## 1. Introduction

T-large granular lymphocytic leukemia is a rare (2% to 5% in Western countries) chronic lymphoproliferative disorder of cytotoxic lymphocytes, usually characterized by an indolent clinical course [1]. According to the World Health Organization (WHO) [2,3], a clonal expansion of large granular lymphocytes (LGLs, 0.5 × 10^9^/L compared to 2 × 10^9^/L previously), persistent for more than six months, is required to diagnose T-LGLL [4]. The median age at diagnosis is 60 years, without sex predilection, even if in women the diagnosis is more often made at a young age [5].

At the time of diagnosis, T-LGLL is often associated with autoimmune disorders and asymptomatic cytopenia, specifically chronic neutropenia, which causes recurrent infection. Despite the B symptoms being rare, they may occur in 20–30% of the cases related to fatigue. Other hematological disorders that can arise in T-LGL leukemia are anemia, including transfusion-dependent anemia (6–22%), pure red aplasia (8–19%) and hemolytic anemia, and thrombocytopenia with a low incidence. Notably, one-quarter of patients have splenomegaly, whereas hepatomegaly or lymphadenopathy are rare [1,6].

Diagnosis is based on (i) cytological identification (blood smear) of LGLs characterized by large size (15–18 μm in diameter) with abundant cytoplasm containing notable azurophilic granules and a round or reniform nucleus with mature chromatin, (ii) flow cytometry of peripheral blood (PB), and (iii) clonality of T-cell receptor (TCR) rearrangement [7,8].

In this report, we described a case of a patient referred to our center with a diagnosis of T-LGLL in 2018, who presented atypical pathological clinical features with the co-existence of two T-cells neoplastic clone, one TCR α/β^+^ and the other TCR γ/δ^+^.

## 2. Material and Methods

In this section, only the analysis performed in our Institute is described (year 2018).

### 2.1. Flow Cytometry

**Figure 1:**
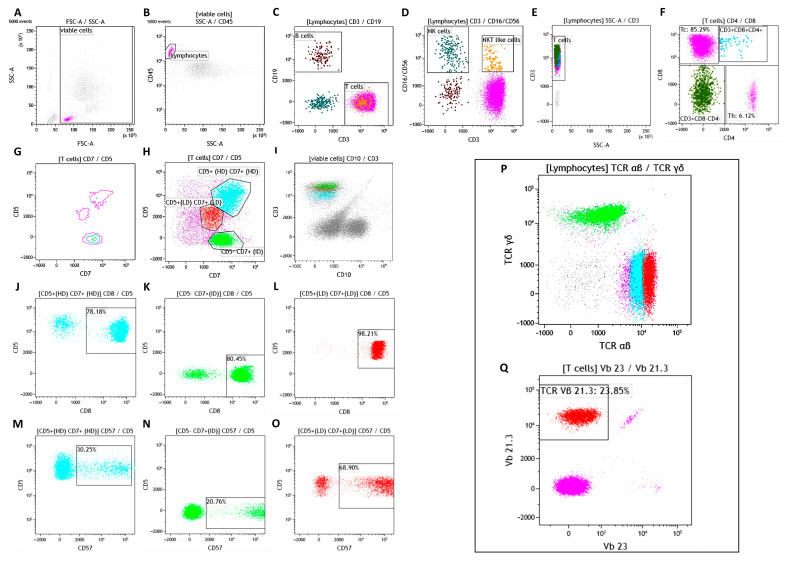
Immunophenotyping analysis of the bone marrow aspirate carried out in our Institute. Any debris, dead cells, and clumps or doublets were excluded by using the FSC-A vs. SSC-A parameters (gate viable cells) (**A**). CD45^+^ lymphocytes (purple gate) were gated on CD45 vs. the SSC-A dot plot (**B**). B-cell lymphocytes, NK cells, NK-T cells, and T cells (red-purple, petrol-green, orange, and purple gates, respectively) were identified from the lymphocytes gate (**C**,**D**). Gating strategy based on CD3 vs. CD8 expression was used to identified different T cells subset (**E**,**F**): Cytotoxic T-cells (Tc, purple gate), CD3^+^CD4^+^CD8^+^ lymphocytes (light-blue gate); CD3^+^CD4^−^CD8^−^ lymphocytes (dark-green gate), and helper T-cells (Th, purple gate). The variable intensity of CD7 vs. CD5 expression (**G**,**H**), gated on T cells, and the CD10 expression (**I**) were used to detect the normal cells (CD5^+HD^CD7^+HD^CD10^−^, cyan gate) and the two pathological populations CD5^−^CD7^+ID^CD10^−^ (green gate) and CD5^+^CD7^+LD^CD10^−^ (red gate), respectively. The (**J**–**O**) dot plots report the percentage expression of CD8 and CD57 vs. CD5 evaluated in the three populations. TCR αβ and γδ expression was evaluated gating lymphocytes population (**P**). The (**Q**) dot plot shows the TCR restriction analysis.

Bone marrow aspirate (BMA) and ascitic liquid sample were evaluated by Flow Cytometry as reported in our previous study of 354 patients [9]. The obtained data were analyzed by using Kaluza Software Version 2.1 (Beckman Coulter, Brea, CA, USA): “rare event”, such as B cells, Natural killer (NK) cells, and NKT-like cells, display function was used favoring the visualization of small subpopulations. For recognition of the smaller subpopulations, cells clustered by specific markers were identified, providing a sufficient denominator of relevant events in the lymphocytes gate (about 15,000 events). An antigen was considered positive when it was at least 20% expressed. In our assays, any debris, dead cells, and clumps or doublets were excluded using forward scatter -Height (FSC-H) by FSC-Area (FSC-A) parameters (Figure 1A, gate “singlets”). CD45^+^ lymphocytes were gated on CD45 versus side scatter dot plot on “singlets” (Figure 1B).

From the lymphocytes gate, B cells (CD19^+^) (Figure 1C), NK cells (CD3^− ^CD16^+^ CD56^+^; Figure 1D), and total T-cells (CD3^+^; Figure 1E) were sequentially identified. From the total T-cells gate, CD8^+^CD3^+^ cytotoxic T-cells (Tc) and CD4^+^CD3^+^ T helper cells (Th) were identified (Figure 1F). Immunophenotyping of pathological Tc and normal T-cells was assessed using a complete panel of T markers: CD7, CD5 (Figure 1G,H), CD10 (Figure 1I), CD57 (Figure 1M–O), TCR αβ, and TCR γδ (Figure 1P). The study of TCR gene repertoire analysis was performed using TCRVβ repertoire kit (IOTest^®^ Beta Mark, Beckman Coulter), showing the clonality of αβ clone (Figure 1Q).

### 2.2. Morphology and Immunohistochemistry (IHC)

Atypical lymphoid cells were detected on BMA smear and May–Grünwald–Giemsa-stained. For morphological analysis, a BM biopsy (BMB), fixed in formalin, decalcified, and paraffin-embedded, was stained with hematoxylin and eosin. When there was no morphological indication of malignant cells, the morphology was considered negative. Positive results were classified as clear lymphoma infiltration or paratrabecular lymphoid aggregation. Non-paratrabecular small lymphoid aggregates were regarded as inconclusive. Moreover, BMB sections were analyzed by using Dako Cytomation Autostainer (Agilent, Santa Clara, CA, USA) and Dako Artisan Staining System with a panel of monoclonal antibodies against B and T cell antigens: anti-CD4, CD8, CD79α, CD20, CD3, and CD57 (Immunohistochemical images of bone marrow biopsy not available.)

## 3. Results

### 3.1. November 2014–February 2018 (Other Institute)

*Clinical history.* This presentation begins with a 55-year-old Caucasian man with severe anemia (hemoglobin value 6 g/dL) and remarkable macrocytosis, not correlated with paroxysmal nocturnal hemoglobinuria (PNH), in November 2014 in another center. (PNH is certainly not the first cause of severe macrocytic anemia. Macrocytosis is mostly caused by alcoholism, vitamin B12 and folate deficiencies, medications, hypothyroidism, or liver disease. Less common etiologies include reticulocytosis, leukemia, myelodysplastic or myeloproliferative syndrome, and primary bone marrow failure syndromes.) The patient’s medical history included acute pancreatitis, cholecystectomy, renal failure, arterial hypertension, cerebral hemorrhage, and absolute lymphocytosis (6600 lymphocytes/mm^3^) developed five years earlier.

*Flow cytometric analyses.* The predominant lymphocytes population in PB was CD3^+^ (98%) of which 86% CD8^+^, 8% CD4^+^, and 7% NK-T. An aberrant T-cell population both in PB (CD3^+^CD8^+^CD2^−^CD5^−^CD7^−^) and in BM aspirate (CD45^+^CD3^+^CD8^+^CD4^−^CD2^+^CD5^+^CD7^+/−^CD57^+^CD10^+^CD1a^−^TCR^+^) was also identified.

*Morphology and IHC*. The PB smear showed small-size granulated lymphocytes, some of these with hand mirror morphology. The preserved granulocytic differentiation and the absence of myeloid and lymphoid blasts in PB had ruled out the hypothesis of acute myeloid leukemia (AML). BMB showed a hypercellular marrow with diffuse atypical lymphoid infiltrate (CD20^+^ in clusters and CD3^++^, CD4^+/− −^, CD8^+^, CD57^+/−^ interstitial) accounting for 80% of the overall cellularity. The erythropoiesis process was reduced, with an excess of E1 and E2 population. The megakaryocytopoiesis and granulocytopoiesis were well represented, with a rise of eosinophils. In addition, splenomegaly and lymphadenopathy (abdominal lymph nodes diameter max 1.5 cm) were revealed.

*Diagnosis, treatment, and progression.* All findings were consistent with the diagnosis of T-LGLL that was confirmed in 2015. Treatment with vitamin B12 and folate was performed without response. Subsequently, a cycle of CHOP (cyclophosphamide + doxorubicin + vincristine + prednisolone) plus methotrexate (MTX) was given but ineffective.

In July 2015, the patient was treated with cyclophosphamide (CPA, 100 mg/day) and prednisone (PDN, 50 mg/day). After 2 months, the therapeutic regimen had to be changed with cyclosporine (CyA), exhibiting a positive clinical response up to June 2017, when the anemia appeared again. Moreover, due to the occurrence of edema and ascites, the chelation and immunosuppressive therapy were suspended. The patient developed chronic hepatopathy (HBV and HCV were negative) and renal failure caused by splenomegaly, which induced squashed of the left kidney. (In our patient case, the only liver disorder investigated is chronic hepatopathy confirmed by serological tests: HBV negative and HCV negative. Although it has been reported that EBV infection can cause HSTCL, the presence of EBV has not been investigated in patient’s serum.)

In October 2017, FC of PB confirmed the presence of a neoplastic T-cell population, with the same immunophenotype seen at diagnosis except for CD2 (now +) and CD5 (now +/−). In addition, CD16 and CD56 expression were also evaluated resulting in CD16^+dim^ and CD56^−^, respectively.

In February 2018, neutropenia, anemia, hypocalcemia, and increase level of creatinine, azotemia, direct bilirubin, alkaline phosphatase, and gamma glutamyl-transferase were observed. As a result of this worsening, treatment with cyclosporine was suspended to be replaced with cyclophosphamide (100 mg/day).

### 3.2. April 2018–June 2018 (Our Institute)

In April 2018, the patient was admitted to our institute.

*Flow cytometric analyses.* FC analysis of BMA showed the presence of two aberrant clones compared to normal Tc population (CD3^+^CD8^+^CD4^−^CD5^HD^CD7^HD^CD57^+^CD10^−^α/β^+^, HD: high density), one α/β and the other γ/δ, accounting for 4% and 7.54% of total viable cells, respectively.

The two pathological clones were easily identified by using the multiparametric flow cytometric acquisition (6 fluorescences) that revealed the different expression intensities only for the two co-expressed surface antigens, CD5 and CD7, compared to normal Tc. In detail, the T-LGL α/β clone was identified as CD5^LD^CD7^LD^ (LD: low density), while the T-LGL γ/δ clone was identified as CD5^−^CD7^ID^ (ID: intermediate density). TCR gene repertoire analysis was performed using FC, revealing that the pathological α/β T cell clone was TCR Vβ 21.3 (encoded by the TRBV11-2 gene) restricted. Specifically, 23.85% of the total T cells were clonal with a frequency ≥10 times higher than its normal limit (mean percentage of expression: 2.38%) [10]. The phenotype data and the gating strategy were reported in Figure 1A–Q. Due to the exacerbation of ascites, a paracentesis was executed. An aliquot of ascitic fluid was analyzed through FC, highlighting the presence of the same neoplastic clones.

**Figure 2:**
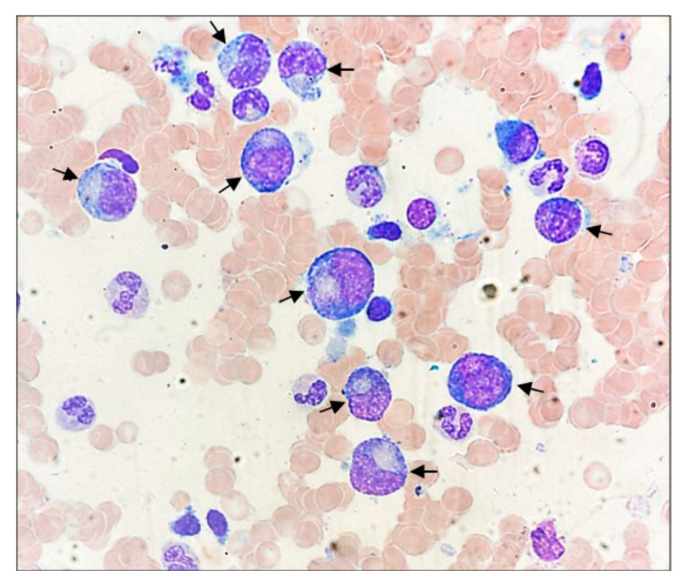
BM aspirate smear. The black arrows highlight the large granular lymphocytes, characterized by medium-large sizes, abundant cytoplasm containing azurophilic granules, and round to reniform hyperchromatic nuclei, with prominent nucleoli (original magnification ×100).

*Morphology and IHC*. Figure 2 reports a representative image of atypical medium-large activated lymphocytes with indented horseshoe (or kidney-shaped) nuclei, copious cytoplasm, and azurophilic granules detected on BMA smear (May–Grünwald–Giemsa-stain).

BMB and BMA revaluation was performed revealing a widespread lymphocytic infiltrate, characterized by medium-large size and abundant cytoplasm, with phenotype CD3^+^CD8^+^CD4^−^CD57^+^CD20^−^CD79a^−^ accounting for 25–30% of the overall cellularity. In detail, the major lymphocyte population (28% of the viable cells) in BMA was CD3^+^CD16/CD56^−^ (96.3%) of which 86.3% CD8^+^.

*Treatment and development*. After a month of CHOP-based chemotherapy, the patient began the palliative care and died in June 2018.

## 4. Discussion

Large granular lymphocyte leukemia (LGLL) is a rare chronic lymphoproliferative disorder characterized by clonal expansion of T or NK cytotoxic cells in the peripheral blood, spleen, and bone marrow. The heterogeneity of clinical, phenotypical, and genotypic features makes diagnosis and treatment difficult [11].

LGL leukemia of mature T cells is the most common subtype and accounts for approximately 85% of cases. T-LGL cells exhibit a post-thymic effector-memory phenotype: CD3^+^TCRαβ^+^CD8^+^CD5^dim^ and or CD7^dim^CD27^−^CD28^−^CD45RA^+^D45RO^−^CD62L^−^CD57^+^ with a variable expression of CD16, CD56, KIRs, and CD94/NKG2, indicating that these cells are late-stage fully differentiated cytotoxic T-lymphocytes [6,11,12]. Although the most common phenotype is CD4^−^CD8^+^, in a few cases a CD4 positive variant has been described, with or without expression of CD8 [4]. In addition, about 10–15% of T-LGLs express TCR γδ that shares several clinical and morphologic features with the more common TCR αβ subtype [13]. In a rare subgroup of patients, a CD4/CD8 double negative phenotype and TCR γδ^+^ have also been reported [4,14].

In this report, we have described a challenging case of T-LGLL that presented to our center with typical as well as rare clinical features. Specifically, the patient had an absolute lymphocytosis, massive infiltration of the leukemic cells in the BM, splenomegaly, and lymphadenopathies. At the onset of the disease, the leukemic cells exhibited the following phenotypes: CD45^+^CD3^+^CD8^+^CD2^+^CD5^+^CD7^+/−^CD57^+^CD10^+^CD4^−^CD1a^−^TCR^+^, which changed during the course of the disease (Table 1).

Specifically, in 2018, a BMA revaluation identified the co-presence of two clones, one αβ and the other γδ, which differed from them in CD5 and CD7 expression levels. Moreover, TCR gene repertoire analysis revealed the Vβ 21.3 restriction of α/β T-cell clones that was described in fewer than 5% of cases documented in the literature [7]. In this rare case, multiparametric and high-sensitivity flow cytometry was effective in discriminating two T-cell clones with different TCR rearrangements as well as varied CD5 and CD7 marker expression intensities. The data demonstrate how flow cytometry is a useful tool in the diagnosis of T-cell lymphoproliferative disorders because it can not only better characterize T-cell neoplasms but also resolve some extremely complicated cases, particularly those involving multiple neoplastic populations.

At the current stage of knowledge, only nine cases with mixed αβ/γδ T-cell LGL clones have been documented, but only for some of them (Table 2) are immunophenotypic (Table 3) features available [15,16,17,18].

Our identification of the mixed αβ/γδ phenotype supports the recent notion by which T-cell repertoire is more dynamic and heterogeneous in T-LGLL [19]. Neff et al. in 4 patients with T-LGLL not only described the simultaneous presence of 2 phenotypically different clones (αβ and γδ), but also their biologic course in response to the therapy showing that the clone switch was correlated with therapeutic success [15]. However, in the absence of TCR αβ and/or γδ expression assessment at diagnosis, in our case we could not advance the hypothesis of clonal T-LGLs drift that would explain the partial and/or no response to the therapy.

In daily practice, a small subset of patients also showed overlapping features between T-LGLL and γδ hepatosplenic T-cell lymphoma, making their diagnosis difficult, especially when splenectomy is not performed, and histology of spleen or liver are not available for analysis [20]. Notably, as reported in two cases by OK et al., a typical misleading situation is the presence of aggressive T-LGLL and HSTCL [21]. HSTCL is a rare and aggressive extranodal type of T-cell non-Hodgkin lymphoma (1%) that usually arises in adolescents and young adults, with a median age of 35 years (male to female ratio is about 9:1) [22,23].

In HSTCL, the lymphoma cells are typically double negative (CD4^−^CD8^−^) even if a subset of cases are CD8^+^, positive for CD3, CD2, CD7, and negative for CD1a and CD5. CD56 is expressed in the majority of cases, whereas CD57 is usually negative [21,24]. Commonly, cancer T cells express the TCR γδ (75%) followed by TCR αβ (25%); however, about 5% are TCR-silent [2].

In Table 4, we summarize the clinical features of T-LGLL and HSTCL compared to our cases.

Yabe et al., comparing the diagnostic criteria of HSTCL and γδ T-LGL leukemia in 42 patients, revealed that features of absence of splenomegaly, lymphocytes with azurophilic granules, and variable expression of CD5, CD8, CD57, granzyme B, and TCR-αβ were significantly more common in γδ T-LGLL that in HSTCL cases [20]. However, our patient showed some clinical and pathological features common to HSTCL diagnosis such as splenomegaly, lymphoma cells expression of TCR γδ, lack of CD5, and elevated alkaline phosphatase [22]. Detection of cytogenetic aberrations are usually useful for differentiating T-LGLL from HSTCL since isochromosome 7q and trisomy 8 are the most prevalent chromosomal abnormalities associated with HSTCL, but atypical for T-LGLL [27]. In our case, the cytogenetic analysis was not performed given that it is not required by clinical practice. Therefore, the co-presence of the two neoplastic disorders could not be established. (It is likely that the two clones identified in this patient case may have originated from a single clone, which is difficult to document based on the patient’s clinical history.)

## 5. Conclusions

The initial splenomegaly, not subsequently detectable due to the abundant ascites, and the aggressive course of the disease led us to suppose that our patient represented a rare case of concomitant T-LGLL (αβ clone) and HSTCL (γδ clone). However, the patient’s rapid worsening made us unable to perform key analysis to validate the potential HSTCL nature of the γδ clone. As a result, the case remains ambiguous, emphasizing the importance of using differential diagnostic approaches (cytogenetic, molecular, and morphological analyses) to distinguish the two malignances, which are frequently overlapping, in order to select the best therapeutic option and enhance the prognosis.

## Figures and Tables

**Table 1:** Immunophenotype and its evolution during the pathological course.

		Antigen													
		TCR	CD3	CD4	CD8	CD2	CD5	CD7	CD10	CD45	CD16	CD56	CD57	CD1a	Vβ Restriction
2014	PB		+		+	−	−	−							
	BM aspirate	+	+	−	+	+	+	+/−	+	+			+	−	
2017	PB		+			+	+/−	−			+ dim	−			
2018	BM aspirate	αβ	+	−	+		LD	LD	−				+ *		Vβ 21.3
		γδ	+	−	+		−	ID	−				+/− **		not applicable

* CD57 expression was evaluated on the entire TCR α/β aberrant population (CD3^+^CD8^+^CD5^LD^CD7^LD^CD10^−^ CD57^+^; 68.9%). ** CD57^+^: 20.7%; CD57^−^: 79.3%.

**Table 2:** Comparison of clinical and biological features between our patient and 7 cases with a mixed αβ/γδ T-cell LGL clones reported in the literature.

	Our Case	Case 1 [15]	Case 2 [15]	Case 3 [15]	Case 4 [15]	Case 5 [15]	Case 6 [18]	Case 7 [17]
**Patient Characteristics and Clinical Findings**								
Age (year)/sex	55/M	79/M	67/F	68/M	72/F	73/F	69/M	47/M
Splenomegaly	Yes	No	No	No	Yes	Yes	Yes	NA
Lymphadenopathy	Yes	NA	NA	NA	NA	NA	NA	NA
Autoimmunity	No	No	No	Polymyalgia rheumatica	Mixed connective tissue disease	Mixed connective tissue disease	Ulcerative colitis; Positive Coombs test	NA
Hepatic disorders	Chronic hepatopathy (HBV neg, HCV neg)	NA	NA	NA	NA	NA	No	NA
Other neoplasm	No	Prostate carcinoma	No	PCN	B-cell lymphoma	CLL; PCN	No	MDS-MLD
**Hematologic findings**								
Anemia	Yes	Yes	No	Yes	Yes	No	Yes	Yes
Hb value (g/dL)	6	10	12.7	10.4	10.1	13	10.9	7.5
Lymphocytosis	Yes	Yes	No	Yes	Yes	Yes	Yes	No
Neutropenia	Yes	No	Yes	No	No	Yes	Yes	Yes
Thrombocytopenia	No	No	No	No	Yes	No	No	Yes
**Morphologic and molecular features**								
BM involvement (%)	80% (2014); 35–40% (2018)	40%	10%	30%	50%	20%	NA	63%
TCR gene rearrangement	Clonal	Clonal	Clonal	Clonal	Clonal	Clonal	No	Clonal
**Therapy and Clinical course**								
Therapy	CHOP + MTX; CPA + PDN; CyA	MTX; CPA + PDN	None	CPA	CPA	PDN + GCSF + HCQ	MTX; Fludarabine; PDN; CyA; Prednisolone	NA
Clinical outcome	Died	No response; progressive cytopenias	Progressive neutopenia	CR for 5 years; then recurrence anemia	CR for 3 years; then recurrence thrombocytopenia and lymphocytosis	PR	CR	NA

Abbreviations: CLL, chronic lymphocytic leukemia; CR, complete response; GCSF: granulocyte-colony stimulating factor; HBV, human hepatitis B virus; HCV, human hepatitis C virus; HCQ, hydroxychloroquine; MDS-MLD, myelodysplastic syndromes with multilineage dysplasia; NA, not available; PCN, plasma cell neoplasm; PR, partial response.

**Table 3:** Comparison of immunophenotypic profile between our patient and 6 cases with a mixed αβ/γδ T-cell LGL clones reported in the literature, where available.

	Antigen												
	TCR	CD3	CD8	CD2	CD5	CD7	CD16	CD57	CD94	KIR Restriction	Vβ Restriction	% Lym	Ref.
Our case	αβ	+	+		dim	dim		+			Vβ 21.3	18.5	
	γδ	+	+		−	+/−		+				26.5	
Case 1	αβ	+	+		−	dim	+	+				77	[15]
	γδ	dim			dim	dim	+	+/−		CD158a		14	
Case 2	αβ	+	+		−	dim	+/−					21	
	γδ	+			dim	+/−	+	+/−	+/−			32	
Case 3	αβ	+	+		dim	+/−	+/−	+/−				85	
	γδ	+			dim	+/−	+	+/−		CD158b		5	
Case 4	αβ	+	+				+/−	+/−			Vβ13.2	11	
	γδ	+		dim	dim	dim	+/−	+				71	
Case 5	αβ	+	+		−		+/−	+/−				67	
	γδ	+	dim		−		+	+				5	
Case 6 (PB)	αβ	+	+				dim	dim				74	[18]
	γδ	+	−				+					16	

**Table 4:** Comparison of clinical and biological features between our case and T-LGLL, HSTCL malignances.

	Case Report (Years)	T-LGLL	HSTCL
**Patient Characteristics and Clinical Findings**			
Age (year)/sex	55/M	45–75/No predilection [8,25]	35/M < F [21,26]
Initial symptoms	No	Fatigue (20–30%), Infection (15–39%), Asymptomatic cytopenia, Autoimmune disorders (15–40%), B symptoms (20–30%) [1,6]	B symptoms, cytopenia [22]
Splenomegaly	Yes (since 2014)	Yes (25–50%) [1]	Yes (97–100%) [22]
Lymphadenopathy	Yes (since 2014)	Yes (very rare) [1]	Non common (15%) [21]
Autoimmunity	No	Yes (15–40%) [8]: Lupus erythematosus, RA, Sjögren’s syndrome, autoimmune thyroid disorders, coagulopathy, and IBM [1,8]	Non common (2–27%) [22]
Hepatic disorders	Chronic hepatopathy (HBV neg, HCV neg; since 2017)	Hepatomegaly (very rare) [1]	Hepatomegaly (40–80%) [22]
Hypertension	arterial (since 2017)	pulmonary artery hypertension [1]	NA
**Hematologic findings**			
Anemia	Yes (since 2014)	Yes (10–30%) [6]	Yes (73–84%) [22]
Hb value (g/dL)	6	<11 (24–40%) [8] <8 (6–22%) [8]	NA
Lymphocytosis	Yes (since 2009)	Yes (50%) [6]	Non common [26]
Neutropenia	Yes (2018)	Yes (50%) [1,8]	Yes (36–85%) [22]
Thrombocytopenia	No	Yes (<25%) [6]	Yes (45–95%) [22]
**Morphologic features**			
PB lymphocytes	Small granulated, some of these hand mirror	Large and granulated [1,6,8,22]	Small, intermediate, or large devoid of azurophilic granules [22]
BM involvement (%)	80% (2014) 35–40% (2018)	<50% [1]	Yes (% NA)
Splenic involvement	NA	Yes	Yes [22]
**Molecular and genetic data**			
TCR gene rearrangement	Clonal	Clonal [21]	Clonal [21]
Genomic features	NA	Gene mutations in JAK/STAT pathway: STAT3 (30–40%), STAT5b (2%) [6,8]	Isochromosome 7q (25–70%) trisomy 8 (8–53%) [22] Gene mutations in JAK/STAT pathway: STAT3 (9–9.5%), STAT5B (31–33%) [26]
**Therapy and Clinical course**			
Therapy	CHOP + MTX (2014), CPA + PDN (2014–2015 and since march 2018), CyA (2015–February 2018), CHOP (April–May 2018)	MTX; CPA (50–100 mg per day, OR), CyA (3 mg/kg per day orally), Purina analogs (second line therapy) [1]	CHOP/CHOP-like regimens; CPA/PDN followed by allogeneic or autologous transplantation [22]
Clinical course	Died (June 2018)	Indolent [1,8]	Aggressive

Abbreviations: Hb, hemoglobin; IBM, inclusion body myositis; JAK/STAT, Janus kinases/signal transducer and activator of transcription; OR, orally; RA, rheumatoid arthritis.

## Data Availability

The datasets used and/or analyzed during the current study are available from the corresponding author on reasonable request.
